# A Bayesian Argumentation Framework for Distributed Fault Diagnosis in Telecommunication Networks

**DOI:** 10.3390/s19153408

**Published:** 2019-08-03

**Authors:** Álvaro Carrera, Eduardo Alonso, Carlos A. Iglesias

**Affiliations:** 1 Departamento de Ingeniería de Sistemas Telemáticos, Universidad Politécnica de Madrid, 28040 Madrid, Spain; 2 Department of Computer Science, City University London, London EC1V 0HB, UK

**Keywords:** argumentation, Bayesian, distributed, fault diagnosis, federation, future Internet, multi-agent system

## Abstract

Traditionally, fault diagnosis in telecommunication network management is carried out by humans who use software support systems. The phenomenal growth in telecommunication networks has nonetheless triggered the interest in more autonomous approaches, capable of coping with emergent challenges such as the need to diagnose faults’ root causes under uncertainty in geographically-distributed environments, with restrictions on data privacy. In this paper, we present a framework for distributed fault diagnosis under uncertainty based on an argumentative framework for multi-agent systems. In our approach, agents collaborate to reach conclusions by arguing in unpredictable scenarios. The observations collected from the network are used to infer possible fault root causes using Bayesian networks as causal models for the diagnosis process. Hypotheses about those fault root causes are discussed by agents in an argumentative dialogue to achieve a reliable conclusion. During that dialogue, agents handle the uncertainty of the diagnosis process, taking care of keeping data privacy among them. The proposed approach is compared against existing alternatives using benchmark multi-domain datasets. Moreover, we include data collected from a previous fault diagnosis system running in a telecommunication network for one and a half years. Results show that the proposed approach is suitable for the motivational scenario.

## 1. Introduction

Telecommunication companies have seen an exponential increase in their activity in the last few decades [1]. As a consequence, telecommunication networks have been continuously growing, both in size, heterogeneity and complexity. The current Internet is based on the premise of a simple network service used to interconnect end systems where relatively intelligent services are running. That simplicity has allowed a massive growth of the network since the beginning of the primitive Internet [2]. However, the management approach followed by network operators in the current Internet is obstructing its evolution. Furthermore, network management is challenging for next-generation networks [3]. The future Internet needs to optimise the use of its resources continuously and recover from problems, faults or attacks transparently for the network operator and without any impact on the services running over it [4]. Thus, future networks need to be more intelligent and adaptive than the current ones, and their management systems need to be embedded in the network itself, instead of being external systems [5].

In those next-generation networks, many different actors will interact dynamically to offer reliable end-to-end services. That diversity of actors (users, sensors, devices or content providers) will make network operation and management very hard for the traditional network management approach [6]. Services deployed on top of those next-generation networks will be considered as one of those actors that will have to cooperate autonomously with other actors to get the expected result [7]. That dynamic, autonomous and complex cooperation among actors is a crucial requirement to get flexible and efficient networks [8]. Moreover, that complexity of the future Internet will bring a high level of uncertainty to management tasks [9]. However, that uncertainty is not only an issue for the future Internet. The current Internet deals with it as exposed by Clark et al. [10]. They estimated that the current Internet is over-dimensioned by a factor of 400% to ensure its performance under almost any conditions. This means that the strategy of the current Internet is to over-size the network to ensure its availability. However, maintaining this strategy in the future Internet would be very inefficient and costly. Indeed, the cost of network management and support has increased drastically in recent years due to the complexity of the network technologies requiring more highly-skilled engineers and administrators and rounding 200 billion dollars [11]. Therefore, uncertainty management coming from complex networks is an essential requirement for any management system of the future Internet [12].

To deal with that complexity, autonomic approaches have been proposed both for computing [13] and networking [14,15,16]. This trend tries to achieve self-management capabilities with a Monitor-Analyse-Plan-Execute (MAPE) control loop [13] implemented by autonomic managers. Those autonomic managers must perform different management tasks, such as self-configuring, self-healing, self-optimising and self-protecting. However, a single isolated autonomic manager can achieve autonomic behaviour only for the resources it manages, which can lead to scalability problems. Many managers must be coordinated to obtain global autonomic management of the network to avoid those problems. That coordination is another critical challenge to get a real autonomic network management approach.

Summarising, autonomic approaches require innovative aspects and mechanisms to enable the desired self-capabilities to govern an integrated behaviour of the future Internet [17]. Those mechanisms are based on the usage of the specific domain knowledge of network engineering, taking into consideration the dynamism and complexity of the supervised systems. The European Telecommunications Standards Institute (ETSI) supports this autonomic approach with a generic reference model for autonomic networking named Generic Autonomic Network Architecture (GANA) [18], which defines a set of desired properties for those autonomic systems. Those desired properties are automation, awareness, adaptiveness, stability, scalability, robustness, security, switchable and federation. Getting all of them in an autonomic management system is challenging for network operators following the traditional management approach. Thus, Laurent et al. [18] defined some enabling concepts and mechanisms for further research to achieve those desired properties in the management systems of the autonomic future Internet. This autonomic approach is supported by the Internet Research Task Force (IRTF). Behringer et al. [19] described the design goals of the autonomic networking in the Request For Comments (RFC) 7575, and Jiang et al. [20] analysed the wide gap for autonomic networking reviewing the current status of the autonomic aspects of current networks. Among others, they identified troubleshooting and recovery as some of the non-autonomic behaviours of the current Internet in the RFC 7576, which motivated us to develop this work in the field of autonomic fault diagnosis of telecommunication networks.

In conclusion, due to the increasing complexity, heterogeneity and consequent high level of uncertainty in telecommunication networks, autonomic fault management is an exciting research field for network operator companies and research institutions [21,22,23]. Accordingly, our motivation when preparing this paper was to improve the current situation concerning the challenges mentioned about the autonomic future Internet. In particular, we are motivated by the fact that there is still a lack of solutions for autonomic fault diagnosis mechanisms.

In this paper, we present an innovative approach for distributed fault diagnosis based on a Multi-Agent System (MAS), which applies argumentation techniques to reach agreements among agents (i.e., autonomic managers). During the argumentation process, agents use Bayesian reasoning to handle the uncertainty inherent to fault diagnosis tasks of complex systems. Moreover, data privacy restrictions and their distributed nature are also considered, enabling the application of the proposed method in federated network domains.

Thus, the main contributions of the presented work are (i) an argumentation framework for fault diagnosis based on Bayesian reasoning and (ii) a coordination protocol to apply that framework in a Multi-Agent System (MAS) for distributed fault diagnosis in federated network domains. This work was based on Chapter 4 of the first author’s Ph.D. thesis [24].

The rest of this paper is structured as follows. Firstly, Section 2 presents some previous work, which has been used as the basis of the method presented in this paper, and discusses other related works in the research field of distributed fault diagnosis for network management. Section 3 presents the multi-agent architecture proposed to carry out the cross-domain diagnostic process. The proposed Bayesian Argumentation Framework (BAF) is defined in Section 4, and the protocol proposed to apply it in an MAS is proposed in Section 5. Next, in Section 6, experimental results are presented, validated and discussed. Finally, Section 7 presents some concluding remarks and a brief discussion on future work.

## 2. Background

The method proposed in this paper is based on a previous work, which consisted of an MAS for fault diagnosis deployed in a real-telecommunication network [25]. In that MAS, the diagnosis process starts with a request made by a human operator, which offers the first evidence of a fault. Then, the system executes a set of tests to collect other relevant information from the network and other third-party systems. Finally, the system infers the most probable root cause of the fault and shows the result to network operators. The diagnosis system was evaluated in a real-life scenario for a specific service provided by Telefónica O2 Czech Republic. The system performance was measured with several Key Performance Indicator (KPIs), which show the acceptance of the diagnosis system by human operators and the reduction of the average incident solution time.

Following the successful system applied initially for fault diagnosis of one specific service, the system was adopted and deployed in parallel to other services and networks. Then, network operators considered that it would be beneficial if those isolated diagnosis systems could share some relevant information and diagnose collaboratively to solve faults. That feature presents some challenges for the previous MAS architecture due to scalability issues. Specifically, those issues were focused on the reasoning technique applied for uncertainty management, which is one of the key features that made the system applicable to that real-life scenario. That reasoning process under uncertainty was performed with a Bayesian inference engine applying a causal model of faults, which relates the cause of the fault and their symptoms. However, as that reasoning process was centralised in one single agent, the complexity of that Bayesian model increased when new systems and services had to be diagnosed by MAS, which made the system robustness decrease, and the causal model maintainability was more costly.

The Bayesian reasoning technique is widely used for fault diagnosis in the literature [26,27] for different kinds of networks, such as high-speed rail networks [28,29], wireless sensor networks [30,31], or optical networks [32,33]. Consequently, some distributed reasoning techniques were explored to solve that scalability issues. Our first attempt was to apply one of the existing techniques for distributed Bayesian inference [34], such as Distributed Perception Network (DPN) [35] or Multiple Sectioned Bayesian Network (MSBN) [36,37]. However, some requirements for applying those techniques in the considered scenario, i.e., a dynamic and complex environment such as a telecommunication network, made them not fully compatible with the deployed MAS architecture. Among other restrictions, the requirement of combining knowledge from different experts or domains is not directly covered by these techniques without generating one centralised model and splitting it later into partial models. This issue reduced the scalability of the final solution. Then, we explored other alternatives, which would offer an extra degree of flexibility and were compatible with the desired features of the future Internet exposed by [18], paying particular attention to the network federation, which would allow different autonomic managers to collaborate when required. In our case, we would like to have several agents that are able to perform a distributed diagnosis process handling the uncertainty inherent in any diagnostic task under several conditions such as data privacy and access restriction, critical aspects in federated domains. Thus, the main requirement for that desired technique is the MAS must be able to perform distributed reasoning under uncertainty with access restriction to some crucial information for the fault diagnosis process.

Therefore, we explored other possibilities to add the desired capability to the system. Concerning the distributed classification task based on MAS, Modi and Shen [38] proposed an approach based on each agent only receiving a subset of the attributes of the classification domain. That means each agent had a subset of attributes, and all agents knew all dataset instances during the training phase of the classifier. This feature could be a scalability issue when the number of instances increases. In contrast, in the PISA framework proposed by Wardeh et al. [39], data were either distributed among the agents, i.e., each agent had its private local dataset. For our case study, we would need a combination of both approaches adding flexibility to allow an agent to have any quantity or subset of variables (i.e., attributes) of the diagnosis causal model. On the one hand, the main issue in a multi-agent distributed task is not the algorithms themselves, but the most appropriate mechanism to allow agents to collaborate, as said by Gorodetsky et al. [40]. In this aspect, the argumentation technique applied the PISA framework [39], providing a satisfactory collaboration mechanism. On the other hand, our diagnosis system deals with uncertain information and would need agents able to discuss those uncertain variables given a complete probability distribution about any unknown attribute of the diagnosis case using the soft-evidence technique [41]. The PISA framework uses absolute values for attributes, which would be an issue in complex and dynamic environments, such as our motivational scenario.

Exploring other argumentative techniques, we found that Dung [42] gave the basis of the mainstream contemporary work. On that basis, Bondarenko et al. [43] proposed the Assumption-based Argumentation Framework (AAF), which extends Dung’s framework and offers more flexibility to generate and process arguments, including assumptions in the problem. However, these theoretical frameworks do not deal with uncertainty or probabilistic statements. In contrast, other recent works explore the application of probabilistic argumentation frameworks [44,45,46] for modelling uncertain logical arguments [47] extracting them from a Bayesian networks [48,49]. With respect to the application of probabilistic reasoning in argumentation processes, we can find in the literature many works from a mathematical point of view, such as the application of a probabilistic approach to modelling uncertain logical arguments [47,50], direct translations from Bayesian approach to argumentation [51], to a philosophical point of view, such as a Bayesian perspective of testimonies and arguments [52,53], or to a legal point of view, such as an argumentation supporting tool [54]. Following these approaches, we propose to extend the previous diagnosis system [25], which applied Bayesian reasoning to infer fault root causes with an argumentative capability. That capability allows agents to dialogue about diagnosis cases, keeping coherence in the distributed reasoning process under some restrictive conditions mentioned previously for federated domains, such as data privacy or access restriction.

## 3. Multi-Agent Architecture for Distributed Fault Diagnosis

This section presents the proposed multi-agent architecture for fault diagnosis in federated domains. In this scenario, every agent manages a specific network domain and is responsible for monitoring and diagnosing its faults. However, in a federated scenario, some of those faults involve different domains. In those cases, agents use the argumentation framework, proposed in Section 4, to carry out a dialogue during the diagnosis process sharing the minimum required information to perform a distributed diagnosis process. Finally, to coordinate that argumentation, they apply the protocol proposed in Section 5.

**Figure 1:**
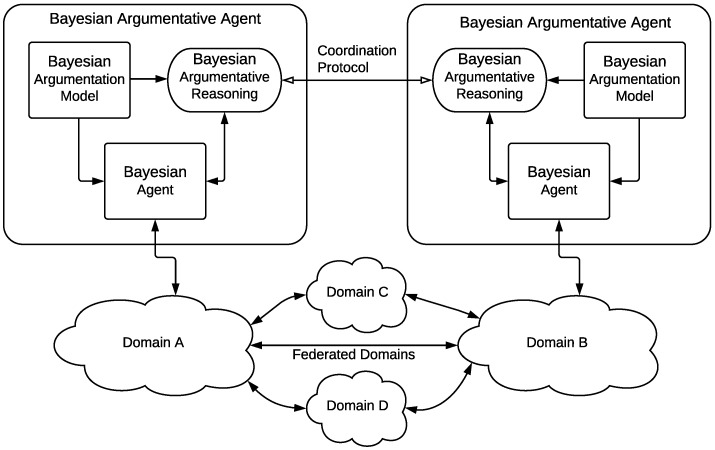
Overview of Bayesian Argumentative agents in federated domains.

In conclusion, we are considering a distributed environment where agents have their partial view of the global problem and cooperate using argumentation techniques to achieve reliable conclusions for a fault diagnosis task in a telecommunication network management scenario. This scenario is schematically depicted in Figure 1, where different network domains are federated to ensure that agents can carry out the inter-domain fault diagnosis process.

The proposed agent architecture, named the *Bayesian Argumentative Agent*, extends the agent architecture presented in a previous works [24,25], labelled as *Bayesian Agent* in the figure. As the *Bayesian Argumentation Framework* and the *Coordination Protocol* are detailed in Section 4 and Section 5, respectively, the following paragraphs presents a brief summary of the *Bayesian Agent* architecture.

The aim of the *Bayesian Agent* architecture is monitoring and diagnosing a specific domain of a telecommunication network. These agents use Bayesian models [55], which provide the capability of modelling causal relations to represent possible faults with their symptoms. The agent collects data in real-time, performs a diagnostic process and offers as output a set of the most probable fault root causes with associated probabilities of occurrence. One of the main features of this agent architecture is the capacity to deal with the uncertainty of complex systems. Another exciting feature of those Bayesian models is that agents can learn by themselves from their experience or based on given knowledge using machine learning algorithms. Thus, every agent has its Bayesian Network (BN), which synthesises its knowledge to infer possible fault root causes based on variables observed from the environment, in our case a telecommunication network. BN is a model that represents the variables involved in the diagnosis process among them and with the possible root causes via a Directed Acyclic Graph (DAG). Therefore, the diagnosis problem domain is described by a set of variables with a set of states in which they can be. Therefore, the problem domain is represented as a causal model; in our case, a Bayesian network. The agent applies this model to discriminate the most probable fault hypotheses. Then, it offers them a conclusion of the fault diagnosis process.

In this work, we are dealing with a distributed scenario with federated domains. We considered agents have their partial perception of the environment, in this case, a portion of the telecommunication network. That means some information could not be accessed directly due to technical issues or privacy restrictions, which generates uncertain situations. Furthermore, data privacy is a critical aspect in network management, which may involve legal clauses, such as final user privacy, to business interests, such as cross-domain actions from different telecommunication operators. Therefore, agents must be able to work under uncertainty and to cooperate keeping data privacy to diagnose faults in a cross-domain scenario. However, every agent has different experience, knowledge and a partial view of the global problem. Thus, different conclusions (or fault root causes) can be inferred. To achieve agreements for a specific fault case in those cross-domains diagnostic processes, we propose the Bayesian Argumentation Framework (BAF), defined in Section 4, and the Coordination Protocol, exposed in Section 5.

## 4. Bayesian Argumentation Framework

This section proposes a Bayesian argumentation framework to discriminate the most probable cause of a fault during a distributed fault diagnosis process in federated domains. We considered that every agent manages its domain and has a partial view of the global problem. This ability to divide the global problem into domains combined with coordination mechanisms ensures the scalability for large-scale systems [56]. Therefore, the coordination mechanism provided by this argumentation framework is required to ensure the scalability of the multi-agent architecture presented in Section 3. As mentioned previously, the model applied during the hypothesis discrimination phase to reason under uncertainty is the *Causal Model*. This model was used to update the hypothesis set every time new observations were collected from the network. The information used as input and output of the *Causal Model* was used in this argumentation framework to build arguments keeping the uncertainty management capability offered by the model. Thus, the argumentation framework exposed in this section required that every agent had a *Causal Model* to build and process arguments.

All in all, the definition of the argumentation framework is presented in Section 4.1. The possible relations that can exist between arguments of this framework are exposed in Section 4.2.

### 4.1. Framework Definition

The proposed argumentation framework relies on the idea of probabilistic statements built using a *Causal Model*. That model is composed of a set of variables and their conditional probabilistic dependencies, as explained in Section 3. Accordingly, we consider that the problem domain for this argumentation framework is described by a set of variables $V=\{v_{1},\ldots ,v_{n}\}$ and a set of states $S=\{s_{1},\ldots ,s_{m}\}$ in which the variables can be. Each variable $v_{i}\in V$ can be in a state $s_{j}\in S$ with a given probability. The set of states a variable $v_{i}$ can be in is denoted by $S_{v_{i}}\subseteq S$ and is defined as the *variable state set*. We define two types of variables: observations, obs, and fault root causes, frc, which compose the set V=obs∪frc. Those observations and fault root causes are modelled as variables of the agent’s *Causal Model*, which allows the agent to infer the probability of a variable is in a given state. That probability represents the agent’s degree of certainty about the state of a given variable, which is the crucial concept to handle the uncertainty of the diagnosis process. In this argumentation framework, we denote that probability as $p\left(i,j\right)=Pr\left(v_{i},s_{j}\right)=\left[0,1\right]$, where $s_{j}\in S_{v_{i}}$. To condense the probabilities of all states of a given variable $v_{i}$, we define a set of probabilities on that variable, as a statement $st_{v_{i}}$. Formally,

**Definition** **1.** *A **statement**$st_{v_{i}}$ is a pair $\langle v_{i},D\rangle$ where $v_{i}\in V$ and D is a set of probabilities p(i,j), which represent the probability of the variable $v_{i}$ being in the state $s_{j}$.*

A statement $st_{v_{i}}$ on a variable $v_{i}$ is coherent if and only if $\forall p\left(i,j\right)\in st_{v_{i}},$∑p(i,j)=1. That means that a statement is coherent if it represents a *probability distribution* for the possible states of the variable $v_{i}$. Formally,

**Definition** **2.** *A **statement**$st_{v_{i}}$ is **coherent**⇔∀p(i,j)∈D∣∑∀p(i,j)=1. Otherwise, $st_{v_{i}}$ is **incoherent***.

We define three different types of statements: *evidence*, *assumption* and *proposal*. On the one hand, *evidence* is based on an observation collected from the network and represents that a variable is in a specific state. As observed directly from the network, we considered that information is certain and cannot be discussed. Formally,

**Definition** **3.** *Given a coherent statement $st_{v_{i}}=\langle v_{i},D\rangle$, $st_{v_{i}}$ is **evidence**$\Leftrightarrow v_{i}\in obs\wedge \exists \langle p\left(i,j\right)\rangle \in D|p\left(i,j\right)=1$.*

On the other hand, an *assumption* represents an unobserved variable. That means the agent cannot gather that information for any reason, such as technical issues or privacy restrictions. Then, an agent can infer this assumption based on the knowledge synthesised in its *Causal Model*. As an assumption is based on background knowledge and is not certain information, this type of statement can be discussed among agents to clarify the state of the variable, as explained in the following sections. Formally,

**Definition** **4.** *Given a coherent statement $st_{v_{i}}=\langle v_{i},D\rangle$, $st_{v_{i}}$ is an **assumption**$\Leftrightarrow v_{i}\in obs\wedge ∄\langle p\left(i,j\right)\rangle \in D|p=1$.*

Finally, a *proposal* represents a hypothesis for the states of a specific variable, which can be a conclusion of the possible fault root cause or a possible clarification for an assumption. Formally,

**Definition** **5.** *Given a coherent statement $st_{v_{i}}=\langle v_{i},D\rangle$, $st_{v_{i}}$ is a **proposal**$\Leftrightarrow v_{i}\in V\wedge ∄\langle o,p\rangle \in D|p=1$.*

To summarise, statements about different variables in the domain are grouped into a *set of statements* to conform arguments. Based on the three types of statements, we define an argument as a triplet of sets of statements: one set for certain information, another set for uncertain information and the last one for proposing conclusions or clarifications. Formally,

**Definition** **6.** *An **argument**arg is a triplet 〈E,A,P〉, where E is the **evidence set** of arg, A is the **assumption set** of arg and P is the **proposal set** of arg.*

In conclusion, this argumentation framework defines three different types of statements, which represent different types of knowledge. Arguments are built as a triplet of sets of statements: evidence set, assumption set and proposal set.

### 4.2. Relations between Arguments

The framework defined in the previous section was proposed to perform hypothesis discrimination tasks among sets of agents in distributed fault diagnosis processes. Thus, agents have to generate and evaluate arguments to try to finish the process with the most reliable diagnosis conclusion. That evaluation process is based on the relations between every pair of arguments as explained below.

To explain the relations between arguments, we define that a pair of agents, $Ag_{i}$ and $Ag_{j}$, can agree or disagree, because they have different background knowledge and different views of the global problem when they are diagnosing in federated domains. Hence, if $Ag_{i}$, generates an argument, $arg_{i}$, and $Ag_{j}$ generates another as response, $arg_{j}$; there can be two main types of relation between those arguments: a **support** relation, if both agents agree, or an **attack** relation, if not. Moreover, there are different types of attacks. However, before starting with the definition of those attack types, we must define the relations of *similarity* and *preferability* between two statements, α and β, generated by two different agents about a specific variable. *Similarity* is used to check the agreement between agents measuring how similar both statements are. If the statements are not similar, we say agents disagree. Then, *preferability* is used to choose one and discard the other, i.e., to know which of the two statements is preferred against the other. These concepts of *similarity* and *preferability* are explained below, in Section 4.2.1 and Section 4.2.2 respectively. Finally, the types of attacks between arguments are exposed in Section 4.2.3.

#### 4.2.1. Similarity

We define *similarity* between statements as a measure of equivalence between them. If two statements are similar enough, we say they are equivalent to the fault diagnosis task. To measure the *similarity* between two statements, we process those statements as *probability distributions* that represent the possible states of the variable $v_{i}$, as defined in Section 4. Thus, the similarity is used to know if two agents agree or disagree about the state of a specific variable, i.e., if their statements are *similar enough* or not. Strictly, two statements are equal if both have equal probabilities for every state of a variable p(i,j). As agents have their private causal models, it is not probable that two statements from different agents have equal probabilities. For that reason, the definition of similarity between statements includes some permissibility to allow that agreement was found with more flexibility, which reduces the number of arguments needed to achieve a reliable conclusion. Moreover, for our fault diagnosis task, we do not need strict equity between statements. Two *similar* statements are a tolerable agreement between agents to continue with the argumentation process.

Therefore, to measure the *similarity* of two statements α,β about the same variable $v_{i}$, we need to apply a **distance function**, Δ, to get a numeric measure, Δ(α,β)∈R, about how similar two statements are between them to know if agents agree or disagree.This similarity can be measured using different distance metrics, such as Euclidean distance, Hellinger distance, Kullback–Leibler distance, J-divergence distance or Cumulative Distribution Function (CDF) distance. For a review of distance metrics between probability distributions, please refer to the work of Koiter [57]. For our fault diagnosis field, we picked the Hellinger distance [58], which offers the following exciting features. Firstly, it can be normalised to bound the metrics in [0,1], which simplifies its processing in contrast with other unbounded metrics, such as Kullback–Leibler distance or J-divergence. Secondly, it does not require any order sequence among the states of a variable, in contrast with CDF distance, which is targeted towards ordinal distributions. Thirdly, it is symmetric, in contrast with others, such as Kullback–Leibler distance. That symmetry is an interesting feature since it does not require any order between statements to measure the distance between them; because *similarity* must be a symmetric measure. Finally, it is more sensitive near zero and one, in contrast with Euclidean distance. That sensitivity is a desirable feature because probabilities near those values in a statement represent that an agent is almost sure that a variable is (p(i,j)≈1) or is not (p(i,j)≈0) in a given state. This feature is suitable because a statement that is *more sure* about the state of a variable should be less similar than other less certain or confident ones.

Then, with the normalised Hellinger distance [58], shown in Definition 7, chosen to measure the similarity between two statements, Δ(α,β)∈R, we define a **threshold** th=[0,1] to establish the bound distance between two statements to be classified as *similar enough*. Therefore, two statements α,β about the same variable $v_{i}$ are *similar enough*, if the distance between them is below the **threshold**, Δ(α,β)<th.

**Definition** **7.** *Given two discrete probability distributions $P=(p_{1},\ldots ,p_{k})$ and $Q=(q_{1},\ldots ,q_{k})$, their normalised Hellinger distance is defined as:*

$$H\left(P,Q\right)=\frac{1}{\sqrt{2}}\sqrt{\sum_{i=1}^{k}{\left(\sqrt{p_{i}}-\sqrt{q_{i}}\right)}^{2}}$$

Based on this definition, a threshold value near zero would imply strict behaviour, because, then, agents only agree when the distance between them is narrow. That behaviour would increase the number of arguments to achieve a conclusion. In contrast, a threshold value near one would entails a permissive behaviour, as agents would almost always agree, which would reduce the duration of the argumentation. However, any convergence would not be achieved. Accordingly, a threshold value between the two bounds should be adjusted depending on the preferences between these two behaviours. In the same sense, a value above 0.5 would have no sense to get agreement at the end of the argumentation, because it would diverge the beliefs of the agents instead to converge to a common conclusion. Thus, the threshold value should be between zero and 0.5 to foster agreements, 0<th<0.5.

Finally, we formally define *similarity* as follows:

**Definition** **8.** *Given two coherent statements α,β about the same variable $v_{i}$, a distance function *Δ* and a threshold th, α is **similar** to β, and vice versa ⇔Δ(α,β)<th. Otherwise, they are not similar.*

#### 4.2.2. Preferability

After presenting the concept of *similarity*, we define *preferability* between two statements as an order of preference between them. As the goal of the system is to diagnose fault root causes, a statement that contains more reliable information about a variable is preferred against others. As mentioned above, when two statements are not similar enough, i.e., they are not equivalent, we can define an order of preference between them.

As mentioned previously, a statement is composed of a set of probabilities p(i,j) that a variable is in a given state. Those probabilities represent the agent’s degree of certainty about the state of a given variable. That means a probability p(i,j)≃1 represents the agent is almost sure the variable $v_{i}$ is in the state $s_{j}$. Contrarily, a probability p(i,j)≃0 means the agents is almost sure the variable is not in that state. For the fault diagnosis task, that certainty is more valuable than other less certain probabilities, such as p(i,j)≃0.5. Thus, we would prefer the statement that represents the highest level of certainty to get more confident conclusions. Notice that this preference is valid because we are considering that all agents have a common goal of diagnosing faults, and they cooperate to achieve it. In competitive environments, every agent could have different preferences, and this decision could be made based on different criteria. However, we are considering only collaborative behaviours in this work. Then, we formally define *preferability* as follows:

**Definition** **9.** *Given two coherent statement α,β about the same variable $v_{i}$ with their respective sets of probabilities $D_{\alpha },D_{\beta }$, α is **preferred** to β$\Leftrightarrow \exists p\left(i,j\right)\in D_{\alpha }|\forall p\left(i,k\right)\in D_{\beta },p\left(i,j\right)>p\left(i,k\right)$.*

Finally, the orderability of statements provided by this preferability property is an interesting feature to solve conflicts and to choose the preferable statement of a set. This property is used in the conflict resolution strategies exposed in Section 5.3.

#### 4.2.3. Types of Attacks

At this point, we have defined two key concepts: *similarity* and *preferability*. Now, we define the different type of attack relations that can exist between two arguments, $arg_{j}$ and $arg_{i}$, based on those key concepts. We define three different **attack** types if agents disagree on a specific type of statement: *discovery*, *clarification* and *contrariness*.

**Definition** **10.** *Given two arguments $arg_{i}$ and $arg_{j}$, generated by agents $Ag_{i}$ and $Ag_{j}$, respectively, if $arg_{j}$ contains any new evidence, $st_{v_{i}}|v_{i}\in obs$, about the diagnosis in progress that is not in $arg_{i}$, we define that $arg_{j}$ is a **discovery** for $arg_{i}$.*

If $arg_{j}$ is a *discovery* for $arg_{i}$, $arg_{i}$ is discarded, and agent $Ag_{i}$ should generate a new argument including the new evidence. The *discovery* is the most basic attack type because it modifies the ground of the reasoning process. Hence, if the ground (the evidences) changes, the output of the inference process (the conclusions) could change as well.

**Definition** **11.** *Given two arguments $arg_{i}$ and $arg_{j}$, generated by agents $Ag_{i}$ and $Ag_{j}$, respectively, when $arg_{j}$ contains a proposal about the variable, $st_{v_{i}}|v_{i}\in obs$, and $arg_{i}$ contains an assumption of that variable, if both statements are not similar and the proposal is preferred to the assumption, we define that $arg_{j}$ is a **clarification** for $arg_{i}$.*

If $arg_{j}$ is a *clarification* for $arg_{i}$, $arg_{i}$ is discarded, and agent $Ag_{i}$ should accept the proposal and generate a new argument including it. A *clarification* attack tries to offer more certain information about an unknown variable represented in the assumption.

**Definition** **12.** *Given two arguments $arg_{i}$ and $arg_{j}$, generated by agents $Ag_{i}$ and $Ag_{j}$, respectively, when $arg_{j}$ contains a proposal that contains a possible conclusion of the diagnosis process, $st_{v_{i}}|v_{i}\in frc$, and $arg_{i}$ contains other possible conclusions, $st_{v_{j}}|v_{j}\in frc$, if both conclusions are not similar enough, we define that $arg_{j}$ is a **contrariness** for $arg_{i}$, and vice versa.*

As no statement is discarded in this attack type, both agents stop arguing until new *discoveries* or *clarifications* appear during the argumentation process. This type of attack is resolved globally at the end of the argumentation process of the coordination protocol, as shown in Section 5.

Finally, we define that an argument **supports** another one if no attack relation exists between them. If a support relation exists among all pairs of non-discarded arguments, a global *agreement* has been achieved. However, as every agent has its domain knowledge synthesised in the *Causal Model*, we cannot ensure a global agreement for any argumentation. Thus, a conflict resolution mechanism must be applied if required.

## 5. Coordination Protocol

This section proposes a coordination protocol for distributed autonomic fault diagnosis in federated domains based on the argumentation framework proposed in the previous section. In this protocol, there are two different agent roles, namely the ***Argumentative*** role and the ***Manager*** role. An *argumentative* agent is responsible to generate and process arguments. A *manager* agent is responsible for several tasks: (i) to establish a coalition of *argumentative* agents to argue; (ii) to decide when an argumentation process has finished; (iii) to figure out the conclusion of the argumentation process.

**Figure 2:**
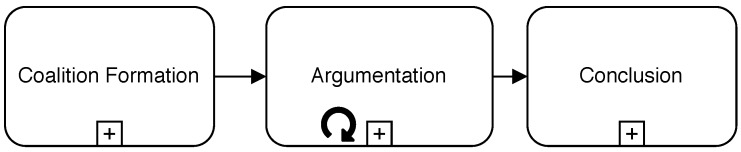
Phases of the Coordination Protocol.

The proposed protocol has three different phases, as summarised in Figure 2. The initial phase for the formation of a group of agents capable of reaching a reliable conclusion for a specific diagnosis case is called the *Coalition Formation Phase*, as explained in Section 5.1. After the argumentation coalition is established and every agent knows the rest of the constituents, the *Argumentation Phase* starts, as exposed in Section 5.2. Finally, when a *manager* agent decides argumentation is finished, all non-discarded arguments are analysed to extract a conclusion during the *Conclusion Phase*, as shown in Section 5.3. For the sake of brevity, a detailed working example of a distributed fault diagnosis is not included in the paper, but it can be found in the Supplementary Material of this article. For the sake of clarity, diagrams included in the following subsections follow the Business Process Model and Notation (BPMN) 2.0 standard specification [59].

### 5.1. Coalition Formation Phase

This phase starts when an *Argumentative* agent (*Initiator agent*) initiates a new process to diagnose an anomaly or a symptom detected in the supervised network elements. This *Argumentative* agent sends a *Coalition Formation Request* message to the *Manager* agent. This message includes data to identify the problem domain. Then, the *Manager* agent broadcasts the message to the rest of the *Argumentative* agents as a *Coalition Invitation* message. When an agent receives that invitation, it decides whether to join the coalition or not. If it decides to join, it must respond to the invitation. Otherwise, it ignores it. This decision is based on the local private knowledge of the agent. In other words, if the agent can offer any relevant information about the problem domain, it would accept the invitation. Otherwise, it would not.

**Figure 3:**
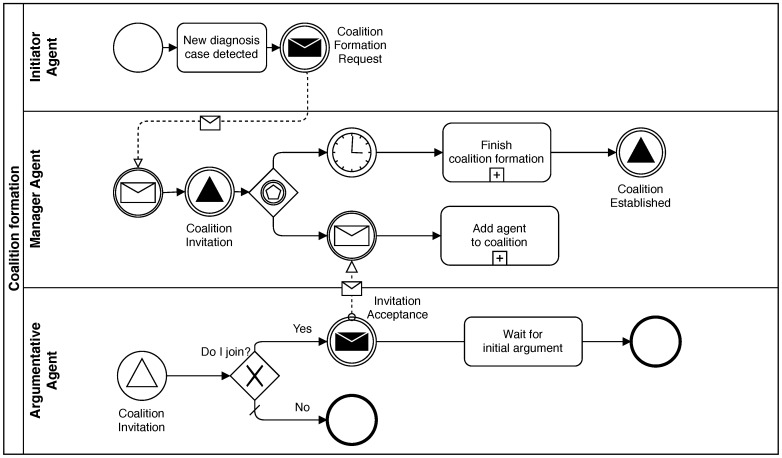
Coalition Formation Phase.

A period of time is specified as the deadline to respond to the invitation to avoid deadlocks while the *Manager* agent is waiting for responses from all *Argumentative* agents. In this way, the *Coalition Invitation* message can be broadcast. After the deadline, the *Manager* agent establishes the coalition with all agents that accepted the invitation and the *Initiator agent*. Finally, it broadcasts a *Coalition Established* message with the complete list of agents that joined the coalition. With this last message, the *Coalition Formation Phase* is finished. Figure 3 shows a diagram of this phase.

### 5.2. Argumentation Phase

This phase starts when the *Coalition Established* message, sent in the previous phase, is received by the *Initiator Agent* who broadcasts the **initial argument** to the coalition. After this step, this agent acts as any other *Argumentative* agent. Later, every agent in the coalition receives that *initial argument* and analyses it to find any attack relation following the reasoning process exposed in Section 4.2. After an argument is processed, the options of an *argumentative* agent are the following: (i) If a *discovery* or a *clarification* relation is found, the agent generates a new updated argument. (ii) Alternatively, if a *contrariness* relation is found, it looks for new information from the environment to add it to the argumentation process. (iii) Finally, if the received argument *supports* the agent beliefs, it can wait until another argument is received.

During this Argumentation Phase, an agent can receive a message that contains an argument while it is processing or generating another one received previously. In that case, the agent should analyse arguments in reception order. Afterwards, it would generate only one argument as the response when all incoming arguments have been processed. This strategy reduces the number of arguments generated during the argumentation dialogue and makes the process require less messaging and, consequently, less computational resources.

**Figure 4:**
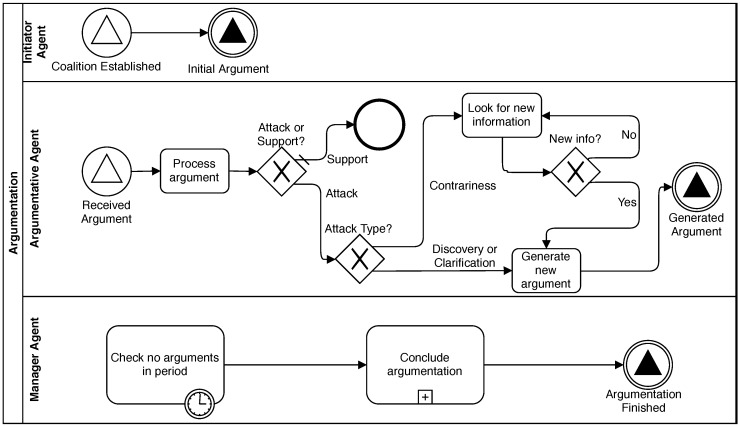
Argumentation Phase.

As shown in Figure 4, agents broadcast any generated argument to all coalition members, including the *Manager* agent. In this way, we ensure that any agent receives all arguments to attack or support any of them during the argumentation dialogue. Every time the *Manager* agent receives an argument, it restarts a timer that is used to know when the argumentation is finished. When all agents remain silent for a time longer than a **silence time-out**, the *Manager* decides this phase is done and starts the *Conclusion Phase*. In other words, the Argumentation Phase continues until every agent has proposed its conclusions to the diagnosis case under consideration, and it does not receive a new argument that makes an agent change those conclusions. It is important to remark that *Argumentative* agents must be able to process and generate arguments in a time lower than the **silence time-out**, to ensure the *Manager* agent does not finish this phase prematurely. Notice that the end of the Argumentation Phase can be delayed in time depending on the number of agents and their configuration. That delay is caused by the impact of the similarity threshold on the agents’ permissibility, as shown in Section 4.2. To avoid this phase being too time-consuming, the *Manager* agent can be configured to allow the coalition member agents to argue during a time, and then, the *Conclusion Phase* starts in any case, even if agents continue arguing. At the deadline, the *Manager* agent sends a message to the coalition members to notify that the argumentation process has finished.

### 5.3. Conclusion Phase

**Figure 5:**
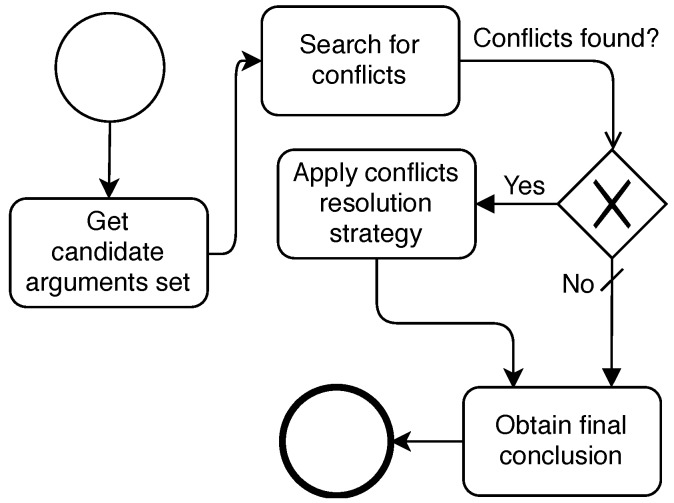
Conclusion Phase.

After the Argumentation Phase is done, the *Manager* agent must process arguments that would have not been discarded due to attacks and extract a conclusion, as shown in Figure 5. We name that set of non-discarded arguments as the **candidate arguments set**. Based on the attack types of the argumentation framework proposed in Section 4, only one type of attack relation can be among arguments of this set: the *contrariness* relation. That is, every argument of the set contains a proposal about a possible fault root cause. If some *contrariness* is found between two arguments, we say that a **conflict** is found. If any *conflict* is found while analysing the *candidate arguments set*, different criteria can be applied to resolve those conflicts and select a conclusion. Contrarily, if no conflict is found, the conclusion all agents agree on is picked as the **final conclusion**. We propose three different conflict resolution strategies for this final phase.

- *Most Popular Conclusion*: This strategy picks as the final conclusion the most popular one in the *candidate arguments set*.
- *Most Confident Conclusion*: This strategy picks as the final conclusion the one with the highest confidence in the *candidate arguments set*.
- *Weighted Conclusion*: This strategy calculates the average confidence value among all arguments with the same conclusion and picks as the final conclusion the one with the highest average confidence value.

Finally, the *Manager* agent sends the conclusions of the distributed diagnosis to all *Argumentative Agents* to notify them that the argumentation is finished including the conclusion of the diagnosis process.

## 6. Results and Discussion

This section presents a report of the experiments performed to assess the validity of the proposed argumentation framework for distributed fault diagnosis. The three main tasks of any fault diagnosis process [60] are symptom detection, hypothesis generation and hypothesis discrimination.

While the previous work [25] addressed the evaluation of the three tasks, this work is focused on evaluating distributed hypothesis discrimination. This task can be considered a classification task that determines the probability of the possible cause being the fault root. Thus, we have evaluated the argumentation as a distributed multi-class classification technique. In this way, the evaluation compares other classification methods in a number of datasets.

The rest of this section is structured as follows. Firstly, Section 6.1 presents the experimentation framework developed to carry out this evaluation process. Section 6.2 summarises the datasets used in the experiments. Finally, Section 6.3 shows the results, and Section 6.4 discusses them.

### 6.1. Experimentation Framework

To provide an empirical assessment of the application of the proposed argumentative framework in the context of standard classification problems, a set of experiments was conducted to compare the results of the proposed technique with other traditional classification techniques. The traditional techniques considered were decision trees (such as J48, Logical Analysis of Data (LAD) Tree (LADTree) or Pruning Rule-based Classification (PART); support vector machines (such as Sequential Minimal Optimization (SMO); simple probabilistic classifiers (such as NBTree); and probabilistic graphs (such as BayesSearch). Most of the considered techniques are available in the WEKA library (Weka Website: http://www.cs.waikato.ac.nz/ml/weka/). In addition, the SMILE library (SMILE Website: http://genie.sis.pitt.edu/ or http://www.bayesfusion.com/) was used because it provides some algorithms not available in WEKA.

Contrary to the mentioned traditional centralised approaches, the proposed argumentative solution requires more than a data mining library to be executed. We developed an experimentation framework that offers an environment to execute agents under federated domains conditions, such as access restriction situations and different background knowledge for every agent. This framework is available as an open-source tool, named Bayesian ARgumentative Multi-Agent System (BARMAS) framework (GitHub public repository https://github.com/gsi-upm/BARMAS). This framework uses the SMILE library as the Bayesian inference and learning engine to enable Argumentative Agents to reason with their *Causal Models* and the MASON simulation framework (MASON Website: http://cs.gmu.edu/~eclab/projects/mason/) as the agent platform.

Notice that the considered traditional classification techniques follow a centralised approach, while the proposed argumentative framework was designed as a distributed solution from the beginning. That is a crucial feature for the application of this work in the motivational scenario, i.e., a distributed fault diagnosis system for federated telecommunication networks. However, it is interesting to compare the results of those techniques for contexts where a centralised one could replace a distributed approach.

Summarising, Table 1 shows the classification techniques considered in the experiments and the respective software libraries used to execute them.

The validation process was carried out with a cross-validation technique with a 10-fold configuration. While for the validation of traditional centralised approaches, 90% of data were used for training and 10% for testing in 10 different iterations, the training data were divided into many sets as argumentative agents were running in the experiment of the BARMAS framework. Therefore, each agent had only a portion of the total training dataset to provide different background knowledge for every agent. For instance, in an experiment with two argumentative agents, each one had only a 45% of the original dataset for training. The other 10% was used for testing. For three agents, 30%; for four agents, 22.5%, etc. To learn from training data, each argumentative agent performed a training process using the *BayesSearch* technique to synthesise the agent’s background knowledge in a *Causal model*. To reproduce access restriction conditions, the set of variables was divided into many subsets as *Argumentative* agents, $V_{1}\cup \ldots \cup V_{n}=V\backslash frc$ (excluding the classification target variables frc). Each agent can access only one of those subsets to reproduce the partial view of the global problem in federated domains.

In addition to the argumentative agents mentioned previously, some extra agents were used in the experiments to reproduce the conditions of the real-life scenario that motivated this work. Firstly, a *Generator* Agent was included in the experiments to generate diagnosis cases. In other words, it simulated the symptom detection task and triggered the distributed classification process. A *Manager* Agent was included to control the argumentation process as explained in Section 5. That *Manager* agentwas configured to follow *the most certain conclusion* strategy, proposed in Section 5.3, as the conflict resolution strategy. The *silence time-out* defined in Section 5.2 was configured to ensure that every agent could generate at least one argument. An *Evaluator* Agent was included to evaluate the conclusion of the argumentation process. This agent was notified by the *Manager* agent when the argumentation process was finished to check the correctness of the classification conclusion. Finally, all *Argumentative* Agents were configured with a *threshold* value equal to 0.2 (th=0.2) to measure the similarity between statements, as explained in Section 4. In conclusion, the following agents were executed in all experiments: *Generator*, *Evaluator*, *Manager*, and a set of *Argumentative* agents.

### 6.2. Datasets

Several public datasets were used for the evaluation, as well as the private one extracted from the case study presented in the previous work [25], which contains fault diagnosis data of a real-life telecommunication network running for one and a half years. Those datasets were used to measure the accuracy of the proposed approach for the multi-class classification problem. Public datasets were collected from the UCI (UCI Repository Website: http://archive.ics.uci.edu/ml/datasets.html) and KEEL (KEEL Repository Website: http://sci2s.ugr.es/keel/datasets.php) repositories and have a meaningful difference among their characteristics to the number of classes, number of instances and number of attributes. An overview of the complexity of the considered datasets is shown in Table 2.

### 6.3. Results

To measure the accuracy of the considered classification techniques, we present the Error Rate (ER) values obtained for every dataset under different uncertainty levels. That uncertainty was generated hiding some variables of datasets to reproduce a crucial aspect of the motivational problem, i.e., uncertainty during fault diagnosis of telecommunication networks. Uncertainty was reproduced in the experiments using missing attributes for the classification algorithms. Three configurations were used to generate different uncertainty levels: no missing attributes (Section 6.3.1), 25% missing attributes (Section 6.3.2) and 50% missing attributes (Section 6.3.3). We considered uncertainty levels above 50% to be quite improbable in real-life scenarios and would result in unreliable conclusions. The following tables show the results of the considered traditional classification algorithms (BayesSearch, J48, LADTree, NBTree, PART and SMO) and the proposed argumentative technique (BARMAS) with different numbers of agents involved in the argumentation process (2, 3 and 4 agents). Notice that the results of different tables should not be compared between them, because the results with no uncertainty were, broadly speaking, better than the results with uncertainty.

Furthermore, to compare different classifiers in this work, statistical tests were applied to the obtained results; concretely, the Friedman test proposed in [61], which is correctly oriented toward the comparison of several classifiers on multiple datasets. This test is based on the rank of each algorithm in each dataset, where the best performing algorithm gets the rank of a score of one, the second-best a score of two, etc. After joining scores for every dataset, the test ranks all classifiers, the best classifier being the one with the lowest score. This test was applied in the three considered scenarios with different uncertainty levels, and other additional tests were performed to evaluate the classifiers under any uncertainty conditions (i.e., testing results for both 25% and 50% in a single Friedman test), shown in Section 6.3.4. Results of these Friedman tests were calculated with an alpha value equal to 0.05 (α=0.05).

#### 6.3.1. Experimentation Scenario: No Uncertainty

Table 3 shows the results with no missing attributes. However, notice that values were truncated, and values equal to 0.00 do not mean a perfect classification. They represent values between 0.00 and 0.01. That implies *Argumentative* agents make no assumptions during the Argumentation Phase because all variables are known with certainty. By analysing these results, we observed that, even with data privacy restrictions and a fully-distributed approach that reduces the number of instances for training by agent, BARMAS are close to traditional techniques. This finding suggests that generally speaking, the use of BARMAS as a distributed approach produces similar results to other centralised alternatives in non-uncertain situations.

However, the Friedman Test showed that, under no uncertainty conditions, the BARMAS approach only statistically improved the BayesSearch algorithm, which indicates that in centralised and non-uncertain scenarios, traditional approaches had slightly better results.

#### 6.3.2. Experimentation Scenario: Moderated Uncertainty

This section exposes the results adding moderated uncertainty to experiments, i.e., removing 25% of the attributes of the classification case as unknown information (missing attributes). That uncertainty allowed BARMAS agents discuss and make assumptions, which was the motivational requirements for this work.

Closer inspection of Table 4 revealed that the difference between columns became significant in uncertain situations for some datasets. For example, focusing on the Mushroom row of Table 4, we observed that *BARMAS* (0.01∼0.02), *BayesSearch* (0.03) and *NBTree* (0.04) (Notice that all of them are Bayesian approaches) had quite low ER compared with other alternatives, such as *J48* (0.26), *LADTree* (0.52) *PART* (0.23) or *SMO* (0.23). Another example can be observed in the Zoo row of the same table. In contrast, all compared alternatives presented similar results in the remaining datasets.

Under moderate uncertainty conditions, the Friedman test showed that the BARMAS approach was one of the alternatives with better results in the ranking.

#### 6.3.3. Experimentation Scenario: Strong Uncertainty

This section shows the considered alternatives under strong uncertainty conditions (50% missing attributes). From Table 5, it is apparent that BARMAS had a lower (or at least equal) error rate than all other alternatives, which attests to the accuracy of BARMAS in situations with high uncertainty (50% missing attributes), being preferable to other alternatives. Furthermore, it offers the flexibility to be applied in distributed environments with private knowledge, as mentioned previously. As the data in Table 5 show, the results for the Zoo or Mushroom datasets presented, again, a significant improvement using Bayesian approaches with differences up to 0.49 for the ER values; as can be seen between *BARMAS* with four agents (0.03) and *LADTree* (0.52) for the Mushroom dataset, shown in Table 5. Contrarily, comparable values are observed (with equal uncertainty levels) in other datasets, such as Solar Flare, Marketing, Nursery, Chess or Network.

Moreover, the Friedman test assessed the BARMAS approach to the best alternative among the considered classifiers under strong uncertainty conditions, which makes its application suitable in the motivational scenario: distributed fault diagnosis tasks in complex and dynamic scenarios, such as telecommunication networks.

#### 6.3.4. Experimentation Scenario: Average Uncertainty

This section presents the scores obtained applying the Friedman test to results from Table 4 and Table 5 together. The objective of this test is to assess the validity of the proposed BARMAS approach under different or undetermined uncertainty conditions, which means that the test offers a ranking for the classifiers in different uncertainty conditions. As shown in Table 6, the benefits of the BARMAS approach are immediately visible under uncertainty conditions.

### 6.4. Discussion

In conclusion, one of the most important consequences of the analysed results was the robustness of the proposed approach against the uncertainty that could be observed focusing on the same dataset of Table 3 and Table 5. For example, at one end, the experiment with four BARMAS agents presented only a 0.02 difference between situations with no uncertainty (ER=0.01) and 50% of missing attributes (ER=0.03) for the Mushroom dataset. In contrast, at the other end, *LADTree* presented a difference equal to 0.52 (ER=0.00 with no uncertainty and ER=0.52 with 50% missing attributes.).

**Figure 6:**
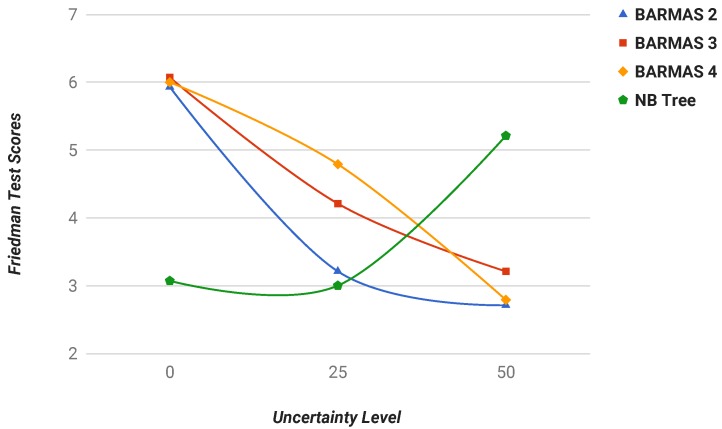
Friedman test scores for the best classifiers by uncertainty level.

Moreover, the Friedman test results showed the improvement of the accuracy under strong uncertainty conditions, as shown in Figure 6, where only classifiers with the best scores are highlighted. Thus, the results of the experiments attest that BARMAS provided a suitable mechanism to perform distributed fault diagnosis under uncertainty in federated domains.

## 7. Conclusions and Future Work

This paper presented an argumentation framework based on Bayesian reasoning for a distributed fault diagnosis task in telecommunication networks. Moreover, a protocol was proposed to apply that framework for a distributed MAS in federated domains. We considered that those agents have different partial views of the global scenario with data privacy restrictions in those domains. Hence, the presented approach proposed an MAS with argumentation capabilities based on Bayesian reasoning for agents with local private datasets. Two agent types were considered in the proposed protocol: *Argumentative* and *Manager* agents. During an argumentation, the protocol allowed a set of *Argumentative* agents to discuss the causes of a detected anomaly, such as a fault in telecommunication networks that must be diagnosed. Those agents interchange arguments that contained information about the diagnosis case until a *Manager* agent decided the argumentation was finished and extracted the conclusion, i.e., the fault root cause of the detected problem.

The proposed method was evaluated with a set of experiments based on empirical data. The obtained results supported its validity as a distributed hypothesis discrimination mechanism for a fault diagnosis system. Among others, we can highlight a set of exciting features for our motivational scenario, i.e., fault diagnosis in telecommunication networks. (i) It allows performing distributed hypothesis discrimination keeping coherence with high robustness against uncertainty. (ii) It allows keeping private knowledge among agents. (iii) It provides a cooperation mechanism for conflict resolution. Furthermore, (iv) it can be deployed in a dynamic and complex environment as agents create temporal coalitions in execution time as required.

After the proposed approach was validated as a distributed hypothesis discrimination mechanism, several possible paths can be explored as future work. An interesting feature that we plan to explore is the usage of trust mechanisms during the argumentation process. Adding reputation, agents can decide if the information received from other agent is more or less reliable than their own beliefs. For instance, if an agent has less experience or is always wrong, its arguments are less reliable than others sent by expert agents, which proposed correct arguments in other past cases. Furthermore, this feature could be completed with feedback mechanisms to check, after the argumentation process, if a statement was true or false, i.e., correct or incorrect. With that feedback, the reputation of any agent could be adjusted at execution time.

Finally, we plan to perform another new set of experiments to look for a set of rules that define the optimal value of the *threshold* parameter based on the context. Thus, agents could have a self-adaptive behaviour depending on the number of agents in the argumentation coalition, the level of uncertainty or the trust they have in the reminder agents to reach the optimal criterion to accept or reject a received argument.

## Figures and Tables

**Table 1:** Summary of the considered classification techniques.

Classification Technique	Acronym	Software Library
J48 - implementation of C4.5 algorithm	J48	WEKA
Logical Analysis of Data (LAD) Tree	LADTree	WEKA
Pruning Rule-based Classification Tree	PART	WEKA
Sequential Minimal Optimization	SMO	WEKA
Naive Bayes Tree	NBTree	WEKA
Bayesian Search	BayesSearch	SMILE
Bayesian ARgumentative Multi-Agent	BARMAS	SMILE + MASON
System		

**Table 2:** Datasets’ summary.

Dataset	# of Instances	# of Classes	# of Attributes
Network (Private dataset with Telefónica O2 Czech Republic rights.)	1183	15	27
Zoo (http://sci2s.ugr.es/keel/dataset.php?cod=69)	101	7	16
Solar Flare (http://sci2s.ugr.es/keel/dataset.php?cod=98)	1066	6	11
Marketing (http://sci2s.ugr.es/keel/dataset.php?cod=163)	8933	9	13
Nursery (http://sci2s.ugr.es/keel/dataset.php?cod=103)	12690	5	9
Mushroom (http://archive.ics.uci.edu/ml/datasets/Mushroom)	8124	2	22
Chess (http://archive.ics.uci.edu/ml/datasets/Chess+%28King-Rook+vs.+King%29)	28056	18	6

**Table 3:** Results without uncertainty (all available data). Best results are marked in bold.

Dataset	BARMAS			Bayes	J48	LAD	NB	PART	SMO
	# of Agents			Search		Tree	Tree		
	2	3	4						
Network	0.16	0.16	0.16	0.18	0.13	0.14	0.14	0.14	**0.12**
Zoo	**0.00**	0.03	**0.00**	0.02	0.02	0.01	0.01	0.02	0.01
Solar Flare	0.29	**0.26**	**0.26**	0.36	**0.26**	0.27	**0.26**	0.29	**0.26**
Marketing	0.71	0.70	0.70	0.71	0.7	0.67	0.68	0.70	**0.66**
Nursery	0.07	0.08	0.09	0.07	**0.01**	0.08	0.03	**0.01**	0.07
Mushroom	0.01	**0.00**	0.01	0.01	**0.00**	**0.00**	**0.00**	**0.00**	**0.00**
Chess	0.5	0.62	0.64	0.61	0.42	0.69	**0.39**	0.46	0.56
Friedman Test	5.93	6.07	6.00	7.50	**3.50**	5.14	**3.07**	4.57	**3.21**
Ranking	6	8	7	9	**3**	5	**1**	4	**2**

**Table 4:** Results with 25% missing attributes. Best results are marked in bold.

Dataset	BARMAS			Bayes	J48	LAD	NB	PART	SMO
	# of Agents			Search		Tree	Tree		
	2	3	4						
Network	0.16	0.17	0.16	0.17	**0.14**	**0.14**	0.15	0.15	0.18
Zoo	**0.09**	0.12	0.11	0.11	0.43	0.4	0.16	0.43	0.41
Solar Flare	0.58	**0.56**	0.6	0.62	0.68	0.67	0.58	0.68	0.79
Marketing	0.70	0.70	0.70	0.71	0.70	0.73	**0.67**	0.70	0.69
Nursery	0.25	0.25	0.26	0.25	**0.24**	0.27	**0.24**	0.26	0.32
Mushroom	**0.01**	**0.01**	**0.01**	0.03	0.26	0.52	0.04	0.23	0.23
Chess	0.64	0.73	0.77	0.68	**0.62**	0.86	0.64	0.68	0.75
Friedman Test	**3.21**	4.21	4.79	5.07	4.71	6.93	**3.00**	6.00	7.07
Ranking	**2**	**3**	5	6	4	8	**1**	7	9

**Table 5:** Results with 50% missing attributes. Best results are marked in bold.

Dataset	BARMAS			Bayes	J48	LAD	NB	PART	SMO
	# of Agents			Search		Tree	Tree		
	2	3	4						
Network	0.22	0.22	**0.21**	0.24	0.23	0.23	0.28	0.23	0.30
Zoo	**0.13**	0.18	0.19	0.16	0.51	0.59	0.28	0.53	0.60
Solar Flare	0.63	**0.61**	**0.61**	0.65	0.69	0.69	0.63	0.69	0.80
Marketing	0.72	0.72	**0.71**	0.72	0.72	0.89	0.73	0.72	0.83
Nursery	**0.27**	**0.27**	**0.27**	**0.27**	**0.27**	**0.27**	**0.27**	0.28	0.32
Mushroom	0.04	0.04	**0.03**	0.05	0.32	0.52	0.22	0.41	0.21
Chess	**0.72**	0.74	0.77	0.74	**0.72**	0.87	0.73	0.74	0.78
Friedman Test	**2.71**	**3.21**	**2.79**	4.43	4.93	7.29	5.21	6.29	8.14
Ranking	**1**	**3**	**2**	4	5	8	6	7	9

**Table 6:** Friedman test scores with average uncertainty levels. Best results are marked in bold.

Dataset	BARMAS			Bayes	J48	LAD	NB	PART	SMO
	# of Agents			Search		Tree	Tree		
	2	3	4						
Friedman Test	**2.96**	**3.71**	**3.78**	4.75	4.82	7.10	4.10	6.14	7.61
Ranking	**1**	**2**	**3**	5	6	8	4	7	9

## Supplementary Materials

The following are available online at https://www.mdpi.com/1424-8220/19/15/3408/s1, Worked Example of the proposed Bayesian Argumentation Framework.

## Abbreviations

The following abbreviations are used in this manuscript:

AAF	Assumption-based Argumentation Framework
MAS	Multi-Agent System
BN	Bayesian Network
FIPA	Foundation for Intelligent Physical Agents
AOSE	Agent-Oriented Software Engineering
BAF	Bayesian Argumentation Framework
DAG	Directed Acyclic Graph
CDF	Cumulative Distribution Function
BPMN	Business Process Model and Notation
BARMAS	Bayesian ARgumentative Multi-Agent System
ER	Error Rate
MAPE	Monitor-Analyse-Plan-Execute
ETSI	European Telecommunications Standards Institute
GANA	Generic Autonomic Network Architecture
IRTF	Internet Research Task Force
RFC	Request For Comments
KPI	Key Performance Indicator
DPN	Distributed Perception Network
MSBN	Multiple Sectioned Bayesian Network
VPN	Virtual Private Network
UPM	Universidad Politécnica de Madrid
