# Supplementary material for: A Bayesian Argumentation Framework for Distributed Fault Diagnosis in Telecommunication Networks
Source: Sensors (Basel). 2019 Aug 3;19(15):3408. doi: 10.3390/s19153408 (PMC6696448; doi:10.3390/s19153408)
Supplement: Supplementary file 1 [file sensors-19-03408-s001.pdf]

Worked Example of the proposed Bayesian Argumentation Framework

Álvaro Carrera, Eduardo Alonso and Carlos A. Iglesias

Universidad Politécnica de Madrid;
a.carrera@upm.es, carlosangel.iglesias@upm.es
City University London;
e.alonso@city.ac.uk

This section presents a worked example to illustrate the operation of a Bayesian ARgumentative Multi-Agent System (BARMAS) in federated scenarios. In this case study, the scenario is composed of several federated networks and an enterprise service is offered across them. This scenario is inspired in the one where the system proposed by Carrera et al. (2014) is running in the Telefónica O2 Czech Republic network. However, this is a simplified version of the real-life scenario considered in the previous work for the sake of clarity.

The rest of this section is structured as follows. Firstly, Section 1 exposes the federated network scenario of this case study where a cross-domain enterprise service is running. Section 2 presents the argumentative agents involved in the case study. Finally, Section 3 exposes a simplified distributed fault diagnosis example using the proposed coordination protocol.

1 Federated Network Scenario

This section exposes a scenario where several telecommunication operators are offering a cross-domain service for international companies. The service allows geographically distributed entities of a company to be connected as if they were physically in the same network (i.e. a kind of Virtual Private Network (VPN) service). In this federated scenario, every operator company manages its network. Under a non-autonomic approach, human operators of every company involved in this cross-domain service should cooperate to handle any possible fault which would happen in the mentioned service. Even though we are considering an autonomic approach, we find the same situation: several agents have to cooperate in carrying out fault diagnosis tasks. Initially, we could consider that this multi-agent approach is not required, that a single fault management system could perform a diagnosis process following the fault diagnosis agent architecture presented in the previous work (Carrera et al., 2014). However, con-

sidering the complexity of Future Internet and other non-technical constraints, such as data privacy or business interests, that is impracticable. Therefore, we consider a federated scenario where Argumentative Agents are responsible for specific domains and cooperate among them to perform cross-domain diagnoses. Figure 1 shows an exemplary agent system deployment in different regions of several European countries. Every agent (presented as blue dots in the figure) is responsible for diagnosing potential faults in its network domain (i.e. in its geographical region).

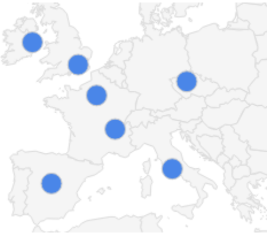

Figure 1: Agent deployment in motivational scenario.

For exemplification purpose, we consider a simplified version of this service. The service under consideration allows geographically distributed entities of a company to be connected as if they were physically in the same network. Then, a set of sophisticated management tasks must be performed. However, we are going to consider a simplified service assuming that only a set of dynamic translations of Internet Protocol (IP) addresses must be done and some registers must be updated with the proper information. Then, we will omit the required low-level configuration tasks. In this simplified scenario, we consider only two offices of a company connected by the described service, one of them in Prague, Czech Republic, and the other one in Madrid, Spain, and that connection is routed through Lyon, France.

2 Deployment of Argumentative Agents

Following the proposed protocol, three different *Argumentative Agents* are deployed in the OSS of their respective cities, and any of them can adopt the *Manager* role if it is required. As each agent can interact with other agents in other diagnosis processes of that service; such as Rome-Paris, Madrid-Berlin, etc.; every agent has its background knowledge based on their own previous experience. In other words, every agent has its *Causal Model* to reason under uncertainty based on their experience of past diagnosis cases. We can name those agents as: *Agent M* (in Madrid), *Agent P* (in Prague) and *Agent L* (in Lyon), as depicted in Figure 2. These agents are monitoring their networks and the interactions among them when the VPN service is running. In this simplified scenario, we consider a translation service running in a server in Lyon (*Agent L* domain) and two registration services running in Prague (*Agent P* domain) and

Madrid (*Agent M* domain). The translation service is the core of this scenario. It is a global IP translation service for many connections of different entities. In contrast, the registration services are two local lists (for Prague and Madrid, respectively) that contain all IP addresses allowed to use the VPN service.

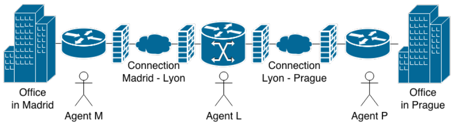

Figure 2: Simplified overview of the example of the federated network scenario.

3 Distributed Diagnosis Example

This section presents a worked example of how a set of three Argumentative Agents performs a distributed fault diagnosis process. For this example, we consider the set of variables V which defines the problem domain and their respective possible states are the ones shown in Table 1[1]. These variables are included in the Causal Model, and they are related to representing the causality relation between symptoms and fault root causes. We consider that all agents have a *similarity threshold* value equals to 0.2, and they calculate it using the Hellinger distance. For further explanations of these concepts, please see the definition of Bayesian Argumentation Framework (BAF).

**Variable**	**States**
SourceMachineUp (SU)	True (T)<br>False (F)
DestinationMachineUp (DU)	True (T)<br>False (F)
SourceIPAddress (SA)	Known (K)<br>Unknown (U)
DestinationIPAddress (DA)	Known (K)<br>Unknown (U)
AllowedIPListsRecentlyUpdated (AR)	True (T)<br>False (F)
TranslationIPListRecentlyUpdated (TR)	True (T)<br>False (F)
FaultRootCause (RC)	AllowedIPListsOutDated (A)<br>DuplicatedIPInTranslationTable (D)<br>WrongTranslation (W)

Table 1: Variables of the Problem Domain for the worked example.

The distributed diagnosis process starts when an anomaly is detected by *Agent P* in the connection between those offices (Prague-Madrid). That anomaly is an unknown source IP address attempting to connect with a server in Prague.

[1]Notice that all arguments exposed below for this worked example use the contracted nomenclature exposed in Table 1 to facilitate the reading.

Then, the **Coalition Formation Phase** starts. It generates the *Coalition Formation Request* message, but no *Manager* agent is known. So, *Agent P* adopts the role of *Manager* agent and sends a *Coalition Invitation* message. After the coalition formation period, two agents (*Agent M* and *Agent L*) have accepted the invitation. Then, *Agent P* sends the *Coalition Established* message to *Agent L* and *Agent M.* Finally, the *argumentation coalition* is established with three constituents, and the protocol continues to the next phase.

At the beginning of the **Argumentation Phase**, *Agent P* generates and broadcasts the initial argument. That initial argument contains the information shown in Argument 1. It has three *evidences* that represent: the source IP address is unknown ({ SA:U }), the destination IP address is known ({ DA:K }), and the destination machine is up and ready to offer its services ({ DU:T }). While those three variables are known with certainty, other set of variables are uncertain and admissible to discuss among all agents. That set is composed by the *assumptions* that represent the uncertainty of the beliefs of *Agent P* as probability distributions. Those probability distributions are inferred using the *Causal Model* of *Agent P*, based on its background knowledge. The output of the inference process offers different probabilities for each variable: if the source machine is up or is down ({ SU:(T=0.7/F=0.3) }), if the list that contains all IP addresses allowed to use the service has changed recently ({ AR:(T=0.85/F=0.15) }) or if the translation service used to route has changed recently ({ TR:(T=0.4/F=0.6) }). Thus, based on the available *evidences* and those *assumptions*, *Agent P* proposes the most probable fault root cause is the list of allowed IP addresses is outdated and that *proposal* is added to the argument as a coherent statement ({ RC:(A=0.7/D=0.05/W=0.25)}).

Argument 1 *Sender: Agent P*
E_{arg_1} →`{SA=U:DU=T:DA=K}`
A_{arg_1} →`{AR=(T=0.85/F=0.15):SU=(T=0.7/F=0.3):TR=(T=0.4/F=0.6)}`
P_{arg_1} →`{RC=(A=0.7/D=0.05/W=0.25)}`

That initial argument is received by the rest of the constituents of the coalition (*Agent L* and *Agent M*). Then, *Agent M* processes that argument getting the *evidences* and comparing its own assumptions with the assumptions sent by *Agent P* in the initial argument. As *Agent M* knows a new evidence useful for this diagnosis case, it increases the *evidence set* with a new piece of information: the list of allowed IP addresses has not been updated recently ({ AR:F }). Then, *Agent M* generates a new argument (Argument 2) with an updated *evidence set*, its own assumptions in an updated *assumption set* and with its own new proposal of the fault root cause in the *proposal set.*

Argument 2 *Sender: Agent M*
E_{arg_2} →`{SA=U:DU=T:DA=K:AR=F}`
A_{arg_2} →`{SU=(T=0.6/F=0.4):TR=(T=0.55/F=0.45)}`
P_{arg_2} →`{RC=(A=0.05/D=0.5/W=0.45)}`

At this point, *Agent L* has received two arguments. It processes them and adds another new evidence: the source machine is up ({ SU:T }). Then, *Agent*

L tries to get information about the status of variable *TR*, but that information is unreachable because the server is overloaded and it is not possible to get that information without stopping the service causing a decrease of Quality of Service (QoS). Hence, that information is not available at diagnosis time and will be handled as an *assumption* during the argumentation. Thus, the updated *evidence set*, the assumption and the proposal of fault root cause of *Agent L* are sent in the Argument 3.

Argument 3 *Sender: Agent L*
E_{arg_3} →`{SA=U:DU=T:DA=K:AR=F:SU=T}`
A_{arg_3} →`{TR=(T=0.45/F=0.55)}`
P_{arg_3} →`{RC=(A=0.05/D=0.7/W=0.25)}`

When *Agent P* receives Arguments 2 and 3, and *Agent M* receives Argument 3, they process them and detect **discovery** attacks between those arguments. So, they accept the new evidences and generate two new arguments: Argument 4 and Argument 5, that contain the beliefs of *Agent P* and *Agent M* respectively. At this point, the *evidence sets* of Argument 3, 4 and 5 contain all available certain information about the diagnosis case exposed in this worked example. Thus, as all agents have sent their beliefs based on the same *evidence set*, they discuss now their assumptions to get the most reliable proposal about the fault root cause.

Argument 4 *Sender: Agent P*
E_{arg_4} →`{SA=U:DU=T:DA=K:AR=F:SU=T}`
A_{arg_4} →`{TR=(T=0.75/F=0.25)}`
P_{arg_4} →`{RC=(A=0.45/D=0.3/W=0.25)}`

Argument 5 *Sender: Agent M*
E_{arg_5} →`{SA=U:DU=T:DA=K:AR=F:SU=T}`
A_{arg_5} →`{TR=(T=0.85/F=0.15)}`
P_{arg_5} →`{RC=(A=0.05/D=0.8/W=0.15)}`

At this point, we summarise the status of the argumentation as follows. Arguments 1 and 2 have been discarded and replaced by Arguments 4 and 5, respectively. Thus, Arguments 3, 4 and 5 represent the beliefs about the most probable fault root cause of agent *L*, *P* and *M*, respectively.

Using the *similarity* and *preferability* concepts defined in BAF, agents detect the statement about the variable *TR* in the assumption set of the Argument 5 ($st_{TR} \in \mathcal{A}_{arg5}$, simplified as $a_{5_{TR}}$) is *similar* to the one in Argument 4 ($a_{4_{TR}}$) [2] and not *similar* to the one in Argument 3 ($a_{3_{TR}}$) [3]. At this point, *Agent M* holds the most *preferred* statement about *TR*. Thus, it generates a new argument (Argument 6) with a proposal for the probability distribution of the variable *TR*.

[2] $\Delta(a_{4_{TR}}, a_{5_{TR}}) = 0.08 < th = 0.2 \Rightarrow a_{4_{TR}}$ and $a_{5_{TR}}$ are *similar*.
[3] $\Delta(a_{3_{TR}}, a_{5_{TR}}) = 0.3 > th = 0.2 \Rightarrow a_{3_{TR}}$ and $a_{5_{TR}}$ are not *similar*.

Argument 6 *Sender: Agent M*
E_{arg_6} →{SA=U:DU=T:DA=K:AR=F:SU=T}
A_{arg_6} → {∅}
P_{arg_6} →{TR=(T=0.85/F=0.15)}

When *Agent P* receives Argument 6, it agrees with the proposal and waits for any other argument. Thus, we say that Argument 6 supports Argument 4.

In contrast, when *Agent L* receives this argument, it finds that Argument 6 is a *clarification* for Argument 3. Thus, *Agent L* adds the received belief as input to the Bayesian inference process as soft-evidence (Pan et al., 2006), discards Argument 3 and sends a new argument with a new proposal inferred based on the updated beliefs (Argument 7). After this, as Argument 6 has achieved its commitment, and it does not contain any proposal about a possible fault root cause, it is discarded too.

Argument 7 *Sender: Agent L*
E_{arg_7} →{SA=U:DU=T:DA=K:AR=F:SU=T}
A_{arg_7} →{TR=(T=0.85/F=0.15)}
P_{arg_7} →{RC=(A=0.05/D=0.9/W=0.05)}

Finally, all available evidences have been discovered, and all agents agree about the possible assumption (only variable *TR* in the example). Only *support* relations (between Arguments 5 and 7) and *contrariness* relations (between Arguments 4 and 5, and between Arguments 4 and 7) exist between arguments. Hence, all agents keep in silence because they do not receive any information that makes them change their beliefs.

After a time in silence longer than the **silence timeout**, *Agent P*, as *Manager Agent*, finishes the **Argumentation Phase** sending a notification to the coalition constituents and starts the **Conclusion Phase**.

Then, *Agent P* filters the set of arguments to get the **candidate arguments set**, that, in this example, is composed by Arguments 4, 5 and 7. So, there are three different proposals:

- *Agent P* proposes $A = 0.45/D = 0.3/W = 0.25$ in Argument 4.
- *Agent M* proposes $A = 0.05/D = 0.8/W = 0.15$ in Argument 5.
- *Agent L* proposes $A = 0.05/D = 0.9/W = 0.05$ in Argument 7.

So, there is a conflict between *Agent P* and the team formed by *Agent M* and *Agent L*. At this point, several strategies can be applied to resolve the conflict, as proposed in the Conclusion Phase of the protocol. For example, let say that the resolution conflict strategy applied is that *the most reliable proposal* is picked as the conclusion. Then, the argumentation concludes when *Agent P* picks that the most reliable fault root cause is $D = 0.9$ (proposed by *Agent L* in Argument 7) that means there is a duplicated IP address in the translation table hosted in the *Agent L* domain. The **argumentation finished** message is sent to all agents in the coalition and the distributed fault diagnosis finishes.

References

Álvaro Carrera, Carlos A. Iglesias, Javier García-Algarra, and Dušan Kolařík. A real-life application of multi-agent systems for fault diagnosis in the provision of an internet business service. *Journal of Network and Computer Applications*, 37:146 – 154, 2014.

Rong Pan, Yun Peng, and Zhongli Ding. Belief update in bayesian networks using uncertain evidence. In *Tools with Artificial Intelligence, 2006. ICTAI '06. 18th IEEE International Conference on*, pages 441–444, 2006.
